# Mitochondrial protein enriched extracellular vesicles discovered in human melanoma tissues can be detected in patient plasma

**DOI:** 10.1080/20013078.2019.1635420

**Published:** 2019-08-27

**Authors:** Su Chul Jang, Rossella Crescitelli, Aleksander Cvjetkovic, Valerio Belgrano, Roger Olofsson Bagge, Karin Sundfeldt, Takahiro Ochiya, Raghu Kalluri, Jan Lötvall

**Affiliations:** a Krefting Research Center, Institute of Medicine, University of Gothenburg, Gothenburg, Sweden; b Department of Surgery and Sahlgrenska Cancer Center, Institute of Clinical Sciences, the Sahgrenska Academy, University of Gothenburg, Gothenburg, Sweden; c Department of Obstretrics and Gynecology and Sahlgrenska Cancer Center, Institute of Clinical Sciences, the Sahgrenska Academy, University of Gothenburg, Gothenburg, Sweden; d Division of Molecular and Cellular Medicine, National Cancer Center Research Institute, Tokyo, Japan; e Department of Cancer Biology, Metastasis Research Center, University of Texas MD Anderson Cancer Center, Houston, TX, USA

**Keywords:** Extracellular vesicles, melanoma, membrane proteins, mitochondria

## Abstract

Extracellular vesicles (EVs), including exosomes and microvesicles, are secreted from all cells, and convey messages between cells in health and disease. However, the diversity of EV subpopulations is only beginning to be explored. Since EVs have been implicated in tumour microenvironmental communication, we started to determine the diversity of EVs specifically in this tissue. To do this, we isolated EVs directly from patient melanoma metastatic tissues. Using EV membrane isolation and mass spectrometry analysis, we discovered enrichment of mitochondrial membrane proteins in the melanoma tissue-derived EVs, compared to non-melanoma-derived EVs. Interestingly, two mitochondrial inner membrane proteins MT-CO2 (encoded by the mitochondrial genome) and COX6c (encoded by the nuclear genome) were highly prevalent in the plasma of melanoma patients, as well as in ovarian and breast cancer patients. Furthermore, this subpopulation of EVs contains active mitochondrial enzymes. In summary, tumour tissues are enriched in EVs with mitochondrial membrane proteins and these mitochondrial membrane proteins can be detected in plasma and are increased in melanoma, ovarian cancer as well as breast cancer.

## Introduction

Extracellular vesicles (EVs) are nano-sized (50–1000 nm in diameter) vesicles with a lipid bilayer membrane that play a significant role in mediating intercellular communication [1,2]. They can do this by either activating surface receptors of recipient cells or by transferring cargo proteins [3], nucleic acids [4], or lipids [5] to recipient cells. The interest in EVs has expanded exponentially over the last five to ten years due to their ability to mediate diverse biological effects, and shuttle molecules between cells. EVs can be found in all body fluids, including blood [6], urine [7], ejaculate [8], and breast milk [9], and they are considered to carry signatures of the cells that produce them. This means that EVs have promising potential as diagnostic markers in disease, especially for cancers. Examples of diagnostic EV markers that have been proposed in cancer to date include glypican-1 protein [10], EpCAM protein [11], KRAS-mutated DNA [12], oncogenic mRNA [13], and microRNAs [13]. However, most EV-based biomarker candidates have initially been identified in cell culture-derived EVs and might not be valid markers for actual human disease. Furthermore, it has been suggested that EV preparations may contain multiple subpopulations of EVs with different surface molecules [14]. We hypothesized that tumor tissues contain EVs that are enriched in subpopulations of EVs that may function as biomarkers. We isolated and characterized EVs directly from human melanoma metastatic tissues and analyzed their surface proteins to identify any subpopulations of EVs. Furthermore, we determined whether or not any such subpopulation is present in plasma from healthy individual *vs* patients with melanoma, ovarian cancer as well as breast cancer.

## Materials and methods

### Human samples

Tissues from melanoma lymph node or skin metastases were obtained from patients who have disease Stage 3 or 4, during surgery and were preserved in complete cell media (without fetal bovine serum, FBS) at 4°C and were used to isolate EVs. A total of 20 ml of peripheral blood was collected from melanoma patients, breast cancer patients, and healthy controls in EDTA tubes. Plasma was obtained by centrifugation at 1880 × *g* for 10 min, followed by a second centrifugation at 2500 × *g* for 10 min. All centrifugations were performed at 4°C. The study was approved by the Regional Ethical Review Board at the University of Gothenburg (096–12), and all participants provided a written informed consent. Plasma from patients with ovarian cancer or benign ovarian cysts was chosen from a previously described cohort collected between 2001 and 2010 [15]. Blood samples were collected after anaesthesia but before surgery. Six ml of blood were collected in EDTA vacutainers using standardized procedures, centrifuged (1000 × *g* for 10 min and 2000 × *g* for 15 min) and directly aliquoted into Eppendorf tubes, frozen and stored at −80°C within 30–60 min after withdrawal. The selected samples had seen one freeze-thaw cycle only. Ethical board approval number are 348–02 and 201–15.

### Cell culture

The human mast cell line, HMC1 cells and erythroleukemic cell line, TF1 cells (ATCC, Manassas, VA) were cultured in IMDM (HyClone, Logan, UT). Human embryonic kidney HEK293T (ATCC, Manassas, VA) and human melanoma cell, MML1 cells were cultured in RPMI1640 (Sigma Aldrich, St Louis, MO) media. Media was supplemented with 10% EV-depleted FBS (Sigma Aldrich), 2 mM L-glutamine (HyClone), and 1.2 U/ml 1-thioglycerol (Sigma Aldrich). The human MSCs from bone marrow were obtained as passage 1 from the MSC distribution of the Institute of Regenerative Medicine at Scott and White, USA, and cultured in alpha minimum essential medium (GIBCO® GlutaMAX™, Invitrogen, Carlsbad, CA) supplemented with 15% EV-depleted FBS (Sigma Aldrich). Three to four passages of MSCs were used for EV isolation. All media contained 100 U/ml penicillin and 100 µg/ml streptomycin (HyClone). For the EV depletion, FBS was ultracentrifuged at 118,000 × *g*_avg_ (Type 45 Ti rotor, Beckman Coulter, Miami, FL) for 18 h and filtered through a 0.22 µm filter as previously described [16].

### Isolation of EVs from melanoma metastases tissue

Subpopulations of EVs were isolated from melanoma metastases using a centrifugation-based protocol. Tumour pieces were gently sliced into small fragments (1–2 mm) and incubated with collagenase D (Roche, Basel, Switzerland) (2 mg/ml) and DNase I (Roche) (40 U/ml) dissolved in RPMI plain medium (Sigma Aldrich) for 30 min at 37°C. After a filtration step (70 µm pore size), cells and tissue debris were eliminated by centrifugation at 300 × *g* for 10 min and 2000 × *g* for 20 min. Supernatants were centrifuged at 16,500 × *g*_avg_ (Type 45 Ti) for 20 min and 110,000 × *g*_avg_ (Type 45 Ti) for 2.5 h to collect larger vesicles and smaller vesicles, respectively. All centrifugations were performed at 4°C. Pellets were resuspended in PBS. Larger and smaller vesicles were combined and further purified by an isopycnic centrifugation using an iodixanol gradient (OptiPrep^TM^, Sigma-Aldrich).

### Isolation of EVs from cell lines

Conditioned media from cell cultures (600 ml) of HMC1, TF1, MML1, HEK293T, and MSCs was harvested and centrifuged at 300 × *g* for 10 min to remove cells. The supernatant was then centrifuged at 2,000 × *g*_avg_ for 20 min to remove apoptotic bodies and cell debris. EVs were pelleted at 16,500 × *g*_avg_ (Type 45 Ti) for 20 min and at 118,000 × *g*_avg_ (Type 45 Ti) for 3.5 h. Pellets were resuspended in PBS. EVs pellets from 16,500 to 118,00 × *g*_avg_ were combined and further purified by an isopycnic centrifugation using an iodixanol gradient.

### Iodixanol density gradient

EVs from tumour tissues in PBS (1 ml) were mixed with 60% iodixanol (3 ml) and laid on the bottom of an ultracentrifuge tube followed by addition of 30% iodixanol (4 ml) and then 10% iodixanol (3 ml). Samples were ultracentrifuged at 178,000 × *g*_avg_ (SW 41 Ti, Beckman Coulter) for 2 h. EVs were collected from the interface between the 30% and 10% iodixanol layers. For the purification of EVs from cell lines, pelleted EVs (1 ml) were mixed with 60% iodixanol (3 ml) and laid on the bottom of an ultracentrifuge tube. A discontinuous iodixanol gradient (35%, 30%, 28%, 26%, 24%, 22%, and 20%; 1 ml each, but 2 ml for 22%) was overlaid. Samples were ultracentrifuged at 178,000 × *g*_avg_ (SW 41 Ti) for 16 h. EVs were collected from the interface of the 20% and 22% iodixanol layers. Collected EVs were diluted with PBS (up to 94 ml) and ultracentrifuged at 118,000 × *g*_avg_ (Type 45 Ti) for 3.5 h. The pelleted EVs were resuspended in PBS.

### Membrane isolation

Isolated EVs were incubated with 100 mM sodium carbonate solution (pH 12) for 1 h at room temperature with rotation. Potassium chloride solution (1 M) was added and further incubated for 1 h. Samples were subjected to iodixanol density gradient purification as described above for EVs, and membranes were collected from the interface between the 30% and 10% iodixanol layers.

## Transmission electron microscopy

One melanoma metastatic tissue from lymph node was acquired during surgery, and the sample was dissected into small pieces. Samples were placed in 150 µm deep membrane carriers (Leica Microsystems, Bensheim, Germany) that were filled with 20% BSA in PBS and high pressure frozen using an EMPactI (Leica Microsystems). A rapid freeze substitution protocol using 2% uranyl acetate (from a 20% methanolic stock solution) in dehydrated acetone for 1 h was then applied [17,18]. The temperature was increased by 3°C/hour to −50°C where samples stayed for the remainder of the protocol, including polymerization. Samples were washed two times with dehydrated acetone before starting infiltration with increasing concentrations of HM20 (3:1, 2:1, 1:1, 1:2, 1:3 acetone:HM20 for 2–3 h each), followed with three changes with HM20 (2 h each and once overnight). Samples were polymerized in UV light for 48 h. Thin sections (70 nm) were cut and contrasted with 2% uranyl acetate in 25% ethanol (4 min) and Reynold’s lead citrate (2 min). For analysis of EVs, a drop (10 µl) of isolated EVs was placed on 200-mesh formvar/carbon copper grids (glow discharged prior to loading of the sample) (Ted Pella, Redding, CA) for 5 min, fixed in 2.5% glutaraldehyde and contrasted in 2% uranyl acetate. Images were obtained using a LEO 912AB Omega 120 kV electron microscope (Carl Zeiss SMT, Mainz, Germany). Digital image files were acquired with a Veleta CCD camera (Olympus-SiS, Münster, Germany).

### RNA isolation and detection

RNA was isolated from melanoma metastases-derived EVs (larger and smaller vesicles) using the miRCURY^TM^ RNA Isolation Kit (Exiqon, Vedbaek, Denmark) according to the manufacturer’s protocol. EV RNA profiles were analyzed using capillary electrophoresis (Agilent RNA 6000 Nano Kit on an Agilent 2100 Bioanalyzer, Agilent Technologies, Palo Alto, CA). A total of 1 µl of RNA was analyzed according to the manufacturer’s protocol as previously described [19].

### Western blot analysis

Proteins from the iodixanol density gradients were separated by SDS-PAGE and transferred to a polyvinylidene fluoride membrane. The membrane was blocked with 5% non-fat dry milk for 2 h and then incubated with anti-CD81 (Santa Cruz Biotechnology, Santa Cruz, CA, sc9158) or anti-MT-CO2 (Abcam, Cambridge, UK, ab91317) at 4°C overnight. After secondary antibody incubation, the immune-reactive signals were visualized using SuperSignal™ West Femto Maximum Sensitivity Substrate (Thermo Fisher Scientific, San Jose, CA) with a VersaDoc 4000 MP (Bio-Rad Laboratories, Richmond, CA).

### Particle measurement

The numbers of particles from the iodixanol density gradients were measured using ZetaView® PMX110 (Particle Metrix, Diessen, Germany). The chamber temperature was automatically measured and integrated into the calculation, and the sensitivity of the camera was set to 80. Data were analyzed using the ZetaView® analysis software version 8.2.30.1 with a minimum size of 5, a maximum size of 5000, and a minimum brightness of 20. Samples were measured in triplicate.

### ELISA

For the direct ELISA, EVs (500 ng per well) were coated on 96-well plates overnight at 4°C. Plates were blocked with 1% BSA in PBS for 1 h and incubated with anti-CD9 (BD Biosciences, San Jose, CA, 555,370), anti-CD81, anti-COX6c (Santa Cruz Biotechnology, sc390414), anti-SLC25A22 (Thermo Fisher Scientific, PA5-29,181), or anti-MT-CO2 antibodies for 2 h. After washing, the appropriate secondary antibodies with HRP were added. The reaction was initiated by adding TMB substrate solution, terminated by 2 M H_2_SO_4_, and the optical density was measured at a wavelength of 450 nm. For the sandwich ELISA, MT-CO2 antibody was coated on black 96-well plates overnight at 4°C. The MT-CO2 antibody was purified on a protein G column prior to use to remove the carrier proteins. Plates were blocked with 1% BSA in PBS for 1 h. EVs or body fluids were added to the wells and incubated for 2 h at room temperature. A total of 50 µl of blood plasma from patients was used. After washing, COX6c antibody was incubated for 1 h and then HRP-conjugated anti-mouse antibody was incubated for 1 h. Luminescent signal was obtained with the BM Chemiluminescence ELISA Substrate (BD Biosciences).

### Isolation of EVs containing MT-CO2 or FACL4

Antibodies against MT-CO2 or FACL4 (Abcam, ab155282) were conjugated to magnetic beads with the Dynabeads® Antibody Coupling Kit (Thermo Fisher Scientific) following the manufacturer’s instructions. Iodixanol-purified EVs were incubated with anti-MT-CO2 or anti-FACL4 antibody-conjugated magnetic beads for 2 h at room temperature with rotation. Unbound EVs were removed and washed with PBS. Bound EVs were eluted with acidic washing buffer (10 mM HEPES, 10 mM 2-(N-morpholino) ethanesulfonic acid, 120 mM NaCl, 0.5 mM MgCl_2_, 0.9 mM CaCl_2_, pH 5) for 10 min.

### LC-MS/MS and protein search

The protein samples were digested with trypsin using the filter-aided sample preparation (FASP) method. Briefly, protein samples were reduced with 100 mM dithiothreitol at 60°C for 30 min, transferred onto 30 kDa MWCO Nanosep centrifugal filters (Pall Life Sciences, Ann Arbor, MI), washed with 8 M urea solution and alkylated with 10 mM methyl methanethiosulfonate in 50 mM TEAB and 1% sodium deoxycholate. Digestion was performed in 50 mM TEAB, 1% sodium deoxycholate at 37°C in two stages: the samples were incubated with 300 ng of Pierce MS-grade trypsin (Thermo Scientific) for 3 h, and then with 300 ng additional trypsin overnight. The digested peptides were desalted using Pierce C-18 spin columns (Thermo Scientific), the solvent was evaporated, and the peptide samples were resolved in 3% acetonitrile, 0.1% formic acid solution for LC-MS/MS analysis. Each sample was analyzed on a Q Exactive mass spectrometer (Thermo Fisher Scientific) interfaced with Easy-nLC 1200 nanoflow liquid chromatography system. Peptides were trapped on the C18 trap column (200 µm X 3 cm, particle size 3 µm), and separated on the home-packed C18 analytical column (75 µm X 30 cm, particle size 3 µm). A gradient from 8% to 24% B over 75 min, from 24% to 80% B over 5 min, and 80% for 10 min (solvent A: 0.2% formic acid, solvent B: 98% acetonitrile, 0.2% formic acid) was used at a flow rate of 200 nl/min. Precursor ion mass spectra were recorded in positive ion mode at a resolution of 70 000 and a mass range of 400 to 1600 m/z. The 10 most intense precursor ions were fragmented using HCD at a collision energy of 30, and MS/MS spectra were recorded in a scan range of 200 to 2000 m/z and a resolution of 35 000. Charge states 2 to 6 were selected for fragmentation, and dynamic exclusion was set to 30 s. Exclusion lists of m/z values of the identified peptides at 1% FDR with a 10-min retention time window were generated from each results file. Subsequently, a second LC-MS/MS analysis was performed using the same settings as before, besides the exclusion of m/z values present in the first LC-MS/MS analysis. The MaxQuant quantification tool with the Andromeda search engine (version 1.5.2.8) was used for the identification and quantification of proteins [20]. Proteins were searched with the following parameters: enzyme specificity, trypsin; variable modification, oxidation of methionine (15.995 Da); fixed modification, carbamidomethylation of cysteine (57.021 Da); two missed cleavages; 20 ppm for precursor ions tolerance and 4.5 ppm for fragment ions tolerance; *Homo sapiens* reference proteome data from Swiss-Prot (20,196 entries); 1% false discovery rate; and a minimum peptide length of seven amino acids. The first major protein identified was chosen as the representative protein of each protein group and was used for further analysis. Normalized label-free quantification intensity of proteins was obtained by a label-free quantification tool, which is implemented in the MaxQuant software, with a minimum of two ratio counts.

### Systematic analysis

Protein localization was obtained from the Uniprot database and only primary localization of proteins was used for the analysis. Biological process terms from Gene Ontology were analyzed using DAVID (https://david.ncifcrf.gov/). The protein–protein interaction network and identification count were obtained from the STRING database (http://string-db.org/) and the EVpedia database (https://evpedia.info), respectively. Heat map was analyzed with Perseus software (http://www.coxdocs.org/doku.php?id = perseus:start).

### ATP synthase activity measurement

The activity of ATP synthase was measured using 10 µg of EVs and MTCO2-EVs with the ATP Synthase Enzyme Activity Microplate Assay Kit (Abcam) following the manufacturer’s instructions.

## Statistical analysis

GraphPad Prism software version 5 (GraphPad Software) was used for *p*-value calculation. The unpaired two-tailed Student’s *t*-test and one-way ANOVA with Turkey’s multiple comparison test were conducted for comparison between two samples and multiple samples, respectively. The unpaired two-tailed Student’s *t*-test with Welch’s correction was conducted for plasma samples. A *p*-value <0.05 was considered to be significant.

## Results

### Isolation and characterization of EVs directly from tumour tissues

**Figure 1.**
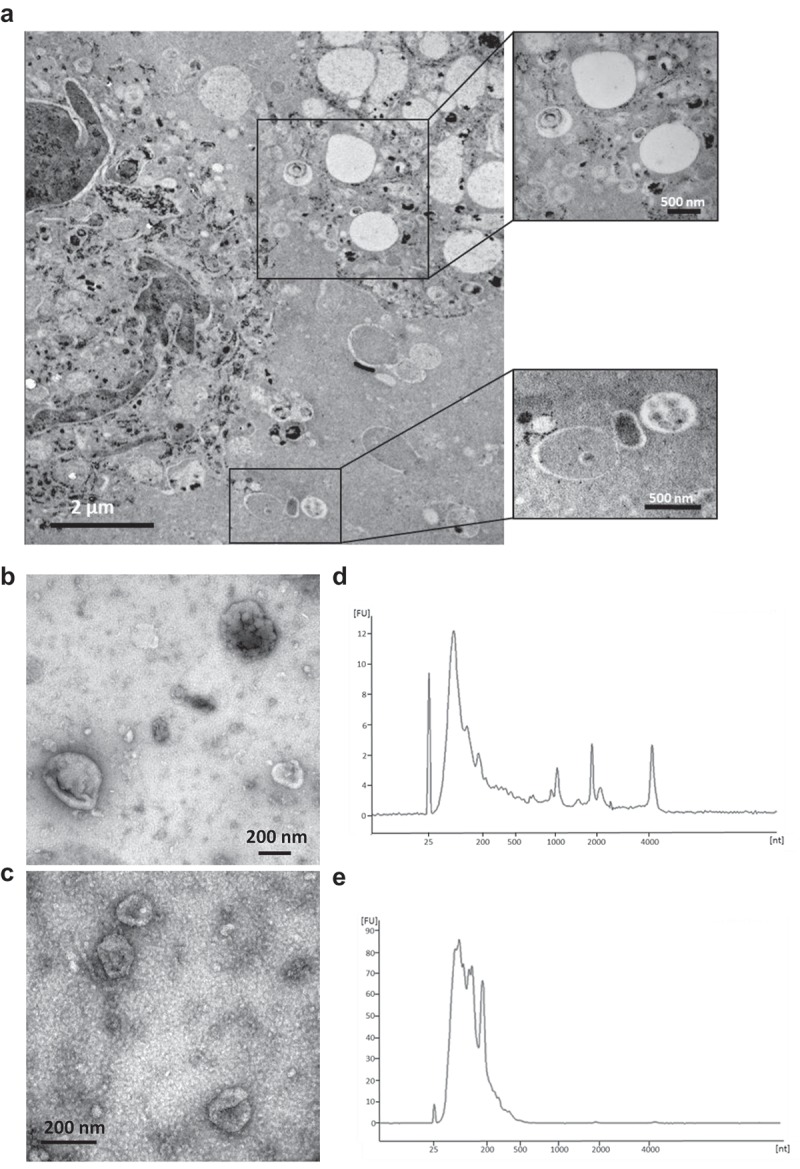
**EVs in melanoma metastases tissue interstitial space were isolated and characterized**. (a) Melanoma metastatic tissue consisted of cells containing melanin accumulations and EVs. Higher magnification pictures showed numerous different types of EVs in the tissue interstitial space. (b-e) Characterization of 16.5k and 118k EVs. Electron microscope images of 16.5k (b) and 118k (d) EVs. The 16.5k EVs have a size range of 100–300 nm, and the 118k EVs have a diameter of approximately 40–100 nm. RNA profiles of 16.5k (c) and 118k (e) EVs. The 16.5k EVs have prominent 18S and 28S ribosomal RNA peaks, whereas the 118k EVs have small RNA with no or very small ribosomal RNA peaks.

First, the existence of EVs in the interstitial space of a melanoma metastatic tissue was visualized by electron microscopy of tumour tissue pieces (Figure 1(a)). Subsequently, melanoma metastatic tissue-derived EVs were isolated using an ultracentrifugation-based protocol that is able to separate EV subpopulations; vesicles are isolated at lower centrifugation speed (16,500 × *g*) and at higher speed (118,000 × *g*). These EV subpopulations were further characterized by electron microscopy and RNA profile analysis and showed typical morphology and RNA profiles of the EV subpopulations “microvesicles” and “exosomes”. The 16.5k vesicles were 100–300 nm in diameter (Figure 1(b), Figure S1) and had an RNA profile comparable to microvesicles (MVs) [21] – a subpopulation of EVs that are considered to be produced by membrane budding – with the presence of 18S and 28S ribosomal RNA peaks and small RNAs (Figure 1(d)). In contrast, the 118k vesicles were 40–100 nm in diameter (Figure 1(c), Figure S1) and exhibited RNA profiles similar to exosomal RNA profiles [21], without prominent ribosomal RNA peaks (Figure 1(e)). Both EVs from tumour tissues contain EV proteins, including Flotillin-1 as well as Calnexin (Figure S2). However, proteomics studies showed that majority of proteins are common in both 16.5k and 118k vesicles, although some proteins are unique in each of these preparation [22,23]. In addition, with current state of art isolation methods, each EV subpopulation is not possible to fully separate from each other. For these reasons, both vesicles were combined for further purification with an iodixanol density gradient, and subsequent proteomic experiments. After iodixanol density flotation, we observed EVs in the isolates that were further processed (Figure S3).

### Identification of melanoma-enriched membrane proteins on EVs

To compare the surface protein expression, EVs that were isolated from five human melanoma metastatic tissues (referred to as MeT1 to MeT5), three cell lines (HEK293T, TF1, and HMC1), and one primary cell (mesenchymal stem cells, MSCs) were treated with high pH solution (200 mM sodium carbonate, pH 12) in order to open the membrane structure as described previously [24]. The high pH-treated membranes were isolated with an iodixanol density gradient after a high salt (1 M KCl) treatment to remove proteins that are ionically bound to the membrane. After this treatment, the removal of dark pigment from pigmented melanoma EVs was clearly observed (Figure S4), confirming that melanin concentration was reduced. The proteins in these membrane-enriched samples were analyzed by mass spectrometry. In total, 2592, 2862, 3461, 2239, 2750, 3577, 2096, 3521, and 1339 proteins were identified from MeT1-, MeT2-, MeT3-, MeT4-, MeT5-, HEK293T-, MSC-, TF1-, and HMC1-EVs, respectively. The identified proteins from different sources overlapped well with the EV proteome database EVpedia [25] (Figure S5(a)). The overlapping percentages for MeT1-, MeT2-, MeT3-, MeT4-, MeT5-, HEK293T-, MSC-, TF1-, and HMC1-EVs were 74%, 79%, 77%, 84%, 76%, 79%, 94%, 83%, and 94%, respectively. These were all significantly higher than randomly selected proteins from the human proteome (38 ± 1.7%) (Figure S5(b)). Similarly, 23.6% and 49.5% of proteins were uniquely found in EVs isolated from human and mice brain, respectively, compared with current EV proteome databases [26], suggesting that tissue-derived EVs were different from cell culture-derived EVs. In addition, classical EV marker proteins such as CD9, CD81, CD63, Syntenin-1, and Flotillins [27] were detected in all EVs with similar abundance, with few exceptions (Figure S6). These results suggest that EVs from tumour tissues are indeed EVs rather than necrotic particles or artefacts of the isolation procedure.

**Figure 2.**
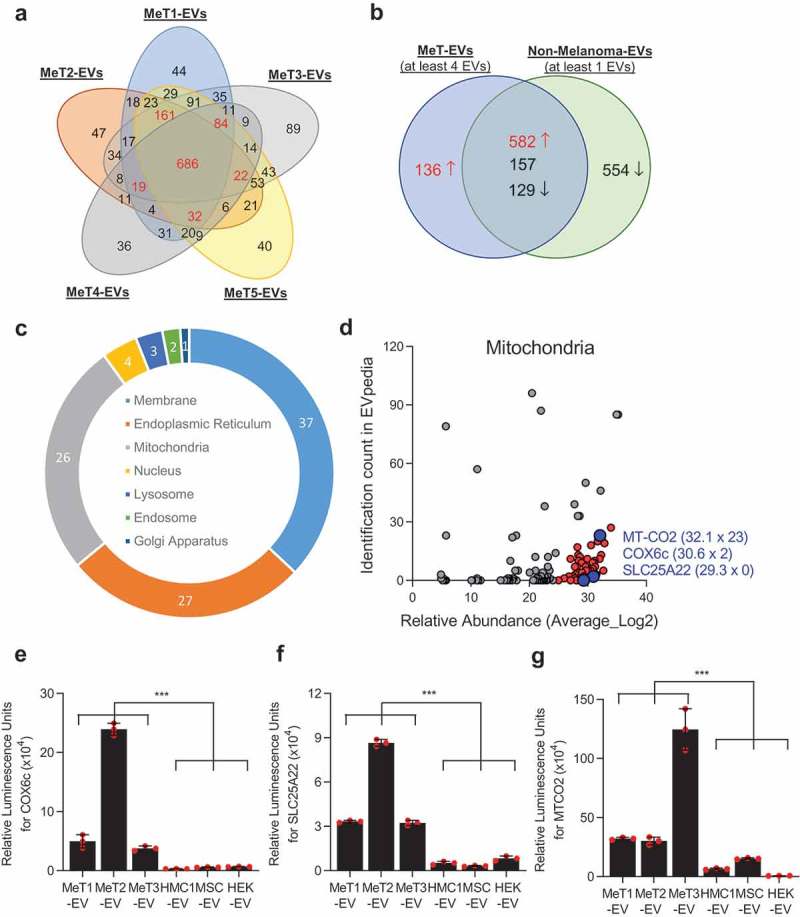
**Proteomic analysis of EVs reveals the existence of mitochondrial membrane proteins**. (a) Only membrane-localized proteins that were identified from five melanoma tissue-derived EVs (MeT1- to MeT5-EVs) were selected and compared. Numbers with red colour are proteins that were identified in at least 4 MeT-EVs. (b) Membrane proteins from MeT-EVs were compared with membrane proteins from non-melanoma-EVs. Common proteins were categorized by 10-fold difference of relative abundance. (c) The sub-cellular localization of 236 candidates was analyzed. The percentage was shown. (d) Mitochondrial membrane proteins were plotted with their relative abundance from a mass spectrometry analysis and their identification count from the EVpedia database. Blue colour is the final three candidates for validation. IC; identification count. (e-g) Three mitochondrial membrane proteins – COX6c (e), SLC25A22 (f), and MT-CO2 (g) – were experimentally validated with direct ELISA. Data are presented as the mean ± SD. ***p < 0.001.

Only membrane proteins that are annotated in the Uniprot database [28] were selected and compared to determine the common surface protein profiles of five different EV populations from melanoma metastatic tissues (Figure 2(a)). Interestingly, membrane proteins from five MeT-EVs were distinct from those from non-melanoma-EVs (Figure S7). In total, 1004 proteins that were identified in at least four MeT-EVs were selected for further analysis. In addition, 1422 membrane proteins that were identified in at least one of the non-melanoma-derived EVs (HEK293T-, MSC-, TF1-, and HMC1-EVs) were selected and compared with membrane proteins from MeT-EVs to find unique membrane proteins on MeT-EVs. One hundred and thirty-six proteins were selected as melanoma-specific surface proteins, as they were not detected in the other EV membrane isolates (Figure 2(b)). In addition, 582 proteins were selected among the 868 common proteins, because these proteins were 10-fold higher in abundance in the MeT-EVs compared to non-melanoma-derived EVs (Figure 2(b)). To increase the confidence of the identification of melanoma specific molecules, the relative abundance of selected surface proteins was taken into account. Finally, 236 proteins that were highly abundant in all 5 MeT-EVs were selected as melanoma-specific surface molecules. (Supplementary Data 1). Surprisingly, the percentage of endoplasmic reticulum and mitochondrial membrane proteins in the membrane protein candidates was higher than 25% (Figure 2(c)). In general, the proportion of mitochondrial membrane proteins (6.9 ± 7.9%) were lower than plasma membrane (74.9 ± 13.2%) when the membrane proteins were analyzed from previous proteomic studies (43 datasets from EVpedia) (Figure S8). Therefore, we focused the current project on the mitochondrial membrane proteins for further characterization, hypothesizing that EVs carrying these proteins exist on a novel subpopulation of EVs that possibly could function as a biomarker in cancer. The relative abundance and the identification count in EVpedia of mitochondrial proteins were plotted (Figure 2(d)). COX6c, SLC25A22, and MT-CO2 – all of which are mitochondrial inner membrane proteins – were selected for validation and tested for their presence in tumour tissue, because they were relatively highly abundant in our samples, and less commonly identified in other EV proteomic studies (Figure 2(d)). These three proteins were highly expressed in melanoma metastatic tissue-derived EVs compared to non-melanoma-derived EVs (Figure 2(e-f)). In addition to mitochondrial membrane proteins, HLA-DR (a plasma membrane protein) and Erlin2 (an endoplasmic reticulum membrane protein) were also highly expressed in melanoma metastatic tissue-derived EVs (Figure S9).

### Existence of mitochondrial proteins on extracellular vesicles

It is known that cancer cells are metabolically active and have enhanced mitochondrial biogenesis [29]. However, mitochondrial genomes are reduced in many cancers, which is considered to support tumour progression [30,31]. Furthermore, whole mitochondria can be transferred between cells in disease conditions such as cancer [32], stroke [33], and lung injury [34], but the details on the mechanism of transfer remain elusive. Recently, MVs from MSCs, a subpopulation of EVs secreted by membrane budding, were reported to contain some mitochondrial components, including proteins and mtDNA [35]. In addition, it has been shown that platelet-derived microparticles can contain intact mitochondria [36]. These findings imply that mitochondrial components are secreted from the cells in the form of EVs. However, it has still not been determined whether mitochondrial proteins are secreted as a subpopulation of EVs. To test this, EVs were isolated from the MML1 and HMC1 cell lines by differential centrifugation coupled with an iodixanol density gradient. The mitochondrial protein MT-CO2, which is translated from the mitochondrial genome [37], was detected at the same density gradient fractions where the EV marker CD81 (Figure S10(a,b)) and most of the particles quantified by NTA are present (Figure S10(c,d)). Surface expression of MT-CO2 as well as the EV markers CD9 and CD81 was detected in EVs isolated from MML1 cells (MML1-EVs) (Figure S10(e)) and HMC1 cells (HMC1-EVs) (Figure S10(f)). However, its expression was higher in MML1-EVs compared with HMC1-EVs (Figure S10(g)), which was consistent with our initial result (Figure 2(g)).

**Figure 3.**
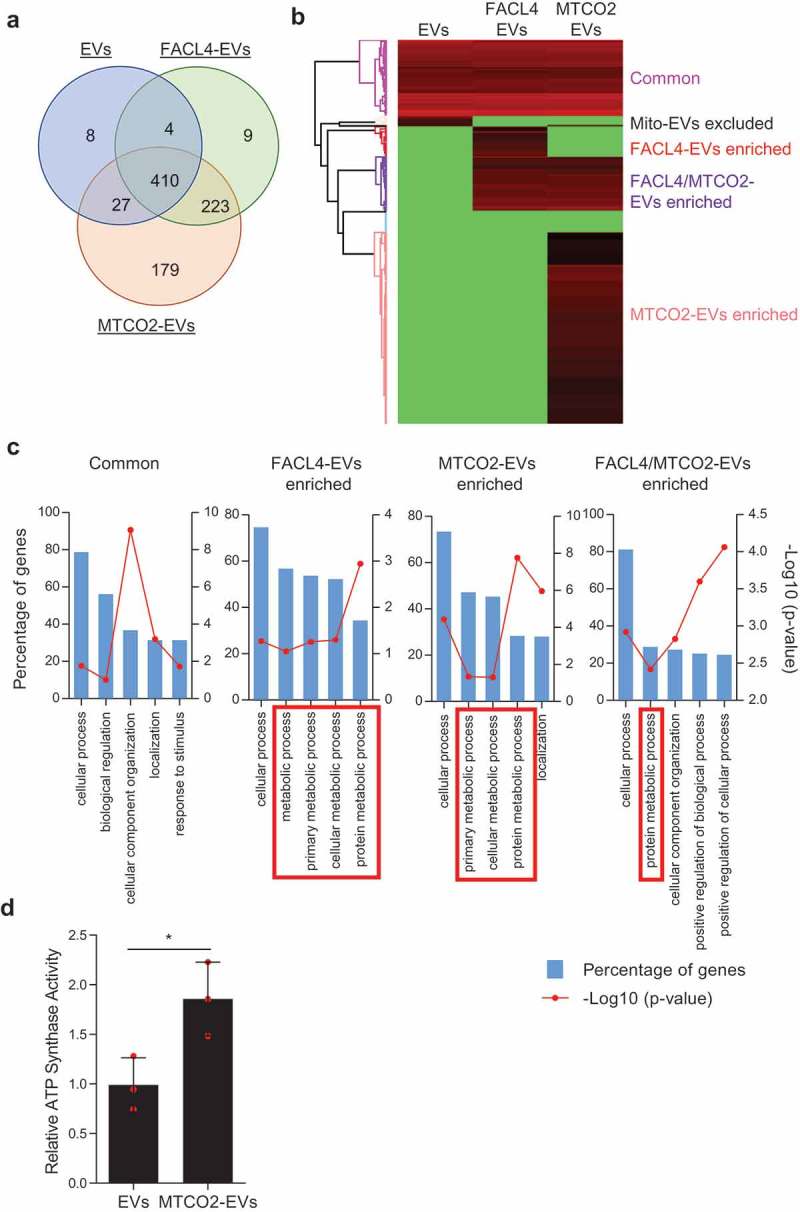
**Subpopulations of EVs harbour mitochondrial proteins**. (a-c) Subpopulations of EVs were isolated with antibodies against FACL4 or MT-CO2, and their proteomes were analyzed. The identified proteins were compared by Venn diagram (a) and heatmap analysis (b). Based on relative abundance of proteins, five different clusters were identified (b), and gene ontology analysis was performed for 4 of the 5 clusters (c). FACL4-EVs and/or MTCO2-EVs clusters enriched with metabolic process-related proteins are shown in red boxes. (d) ATP synthase activity was measured for EVs and MTCO2-EVs. Data are presented as the mean ± SD. *p < 0.05.

The subpopulation of EVs that contain MT-CO2 (MTCO2-EVs) were further isolated from crude EV preparations, and their protein profile was determined by mass spectrometry. EVs that contain FACL4 (FACL4-EVs), another mitochondrial membrane protein that is translated from the nuclear genome, were used as an additional control. In total, 449, 646, and 839 proteins were identified from EVs, FACL4-EVs, and MTCO2-EVs, respectively (Supplementary Data 2). A Venn diagram showed that 410 proteins were common for all three groups, and 179 proteins were uniquely identified in MTCO2-EVs (Figure 3(a)). Heatmap analysis based on the relative abundance revealed that each sample has a distinct proteome profile (Figure 3(b)), and these proteins were categorized into the following five clusters: “Common”, “Mito-EVs excluded”, “FACL4-EVs enriched”, “FACL4/MTCO2-EVs enriched”, and “MTCO2-EVs enriched”. Importantly, most of the classical marker proteins such as tetraspanins, TSG101, Alix, Annexins, and Flotillins are present in all EV populations studied (Supplementary Data 3), implying that there may be a crosstalk between mitochondria and the EV biogenesis pathways. Further, gene ontology analysis showed that FACL4-EVs and MTCO2-EVs clusters were enriched with proteins involved in metabolic processes (Figure 3(c)). In addition, protein–protein interaction network analysis (STRING database) showed that mitochondrial proteins, including ATP synthase subunits in the EVs were interrelated to each other (Figure S11), and the activity of ATP synthase was higher in MTCO2-EVs compared to non-separated EVs (Figure 3(d)). These results argue that subpopulations of EVs contain active forms of mitochondrial proteins.

### Detection of mitochondrial membrane proteins in patient plasma

**Figure 4.**
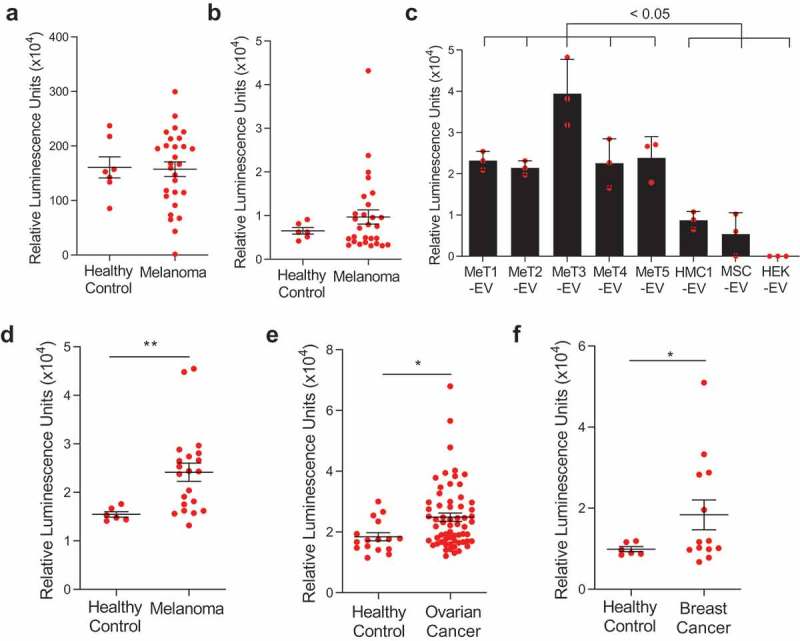
**Mitochondrial membrane proteins on the surface of EVs are unique biomarkers for cancer**. (a,b) The levels of mitochondrial inner membrane proteins, MT-CO2 (a) and COX6c (b) were examined in plasma healthy and melanoma patients. (c) The sandwich ELISA system was validated with melanoma tissue-derived EVs and non-melanoma-derived EVs. Data are presented as the mean ± SD. (d-f) The levels of mitochondrial proteins (MT-CO2 and COX6c) were examined in blood plasma from melanoma patients (*n* = 21) (d), ovarian cancer patients (*n* = 62) (e), and breast cancer patients (*n* = 13) (f). Healthy control was six. Whiskers show the minimum-maximum and lines inside box represent the median. *p < 0.05, **p < 0.001.

We have shown that mitochondrial proteins are present on the surface of the subpopulations of EVs and are more abundant in melanoma-derived EVs. Interestingly, more than 50% of melanoma-specific membrane proteins that are identified in this study was found in plasma EV proteome from health individuals (Figure S12(a)) [38] and 25% of overlapping proteins, including MT-CO2 and COX6c, are mitochondrial proteins (Figure S12(b)). To detect those mitochondrial inner membrane proteins in the circulation, lipid particles were captured by lipid-based capturing system [39] with streptavidin-biotin PEG cholesterol from plasma of healthy and melanoma patients, then MT-CO2 and COX6c levels were evaluated by their antibodies (Figure S13(a)). High luminescence signals for MT-CO2 were detected both in healthy and melanoma patients (Figure 4(a)), but luminescence signals from COX6c were elevated in melanoma patients compared to healthy control (Figure S4(b)). These data suggest the presence of mitochondrial inner membrane proteins in the circulation, associated with lipid particles such as EVs. Next, to determine colocalization of mitochondrial membrane proteins and other membrane-originated proteins, we developed a sandwich ELISA detection system with MT-CO2/COX6c, MT-CO2/CD9, and MT-CO2/RPN1 (Figure S13(b)). This sandwich ELISA was first tested with purified EVs from melanoma tissues and different cell lines. Elevated luminescence signals of MT-CO2/CD9 were detected in both melanoma tissue-derived EVs and non-melanoma cell-derived EVs (Figure S14(a)). However, the signals of MT-CO2/RPN1 were not detected in any EVs (Figure S14(b)). Interestingly, high luminescence signals of MT-CO2/COX6c were observed only in melanoma tissue-derived EVs (Figure 4(c)), which is in line with our proteomics and direct ELISA results (Figure 2(e-g)). These data suggest subpopulations of EVs harbour mitochondrial inner membrane proteins on their surface. In addition, we could identify the presence of MT-CO2/COX6c signals directly in plasma from melanoma patients, without any preceding EV isolation procedure. Importantly, we detected significantly higher levels of combined MT-CO2 and COX6c signal in plasma of melanoma patients compared with healthy controls (*p* = 0.0038) (Figure 4(d)). To test whether this increased concentration of MT-CO2/COX6c in plasma is specific for melanoma, we applied the same assay to plasma from ovarian and breast cancer patients. To our surprise, the concentration of MT-CO2/COX6c was increased in the plasma from both patients with ovarian (*p* = 0.0220) (Figure 4(e)) and breast cancer (*p* = 0.0408) (Figure 4(f)), with significant differences *vs* healthy controls observed. However, there were no significant differences in the concentration of these membrane proteins between patients with malignant *vs* benign ovarian cysts, because those with the benign disease also had some increase of MT-CO2/COX6c (data not shown). Overall, these findings suggest that mitochondrial inner membrane proteins are released and colocalized in plasma in several malignant diseases of different cellular origins, including melanoma, ovarian cancer, and breast cancer. Because both MT-CO2 and COX6c are membrane proteins, we hypothesized that the luminescent signal seen in our sandwich ELISA system is indeed associated with membrane structures such as EVs. Further exploration of the presence of mitochondria-derived EVs in other types of tumours, both malignant and benign, is warranted.

## Discussion

Liquid biopsies are considered to putatively be excellent tools to monitor diseases such as cancer, as they may provide a non-invasive method to monitor disease progression or relapse, as well as treatment response [40]. Recently, EVs have been acknowledged to be advantageous over the current circulating cells and DNA, as they are increased in the circulation in cancer patients [41]. EVs are also known to be intricately involved in tumour progression [42], metastasis [43], and angiogenesis [5] by shuttling their cargo from cancer cells to other cells. The cancer EVs that are secreted into the interstitial space of tissues may leak into the circulation, where they may be quantified or characterized. Numerous studies have attempted to find EV-based protein biomarkers from body fluids of patients including blood or urine. However, the EVs in circulation are dominated by non-cancer cell produced EVs and may hinder successful identification of any biomarkers. Here, we hypothesized that the tumour interstitial space accumulates tumour-derived EVs and may be a better source for biomarker discovery. Isolation of EVs from mouse brain tissues has been reported previously [44], but our study is the first study to isolate EVs from human patient melanoma tumour tissues. In addition, we have applied the membrane proteomics to identify the surface markers, as these may identify relatively distinct subpopulations of EVs [45]. Although EVs are known to carry multiple membrane proteins, more than 60% of EV proteins are non-membrane proteins according to EVpedia [25]. By applying membrane proteomics, we were here able to identify a series of novel EV-membrane proteins, that putatively could be easily quantified by monoclonal antibody assays.

Interestingly, our current data show that melanoma tissue-derived EVs are enriched in mitochondrial membrane proteins, compared with non-melanoma-derived EVs. Furthermore, our study shows that the EVs carrying mitochondrial membrane proteins and have a distinct protein profile, and therefore seem to represent a subpopulation of EVs. The release of mitochondrial components to the extracellular space could be related to enhanced mitochondrial function in cancer, since mitochondria are closely connected with cancer progression, signalling, and cell survival [30,31]. It has been described that mitochondria can releasee vesicles inside of cells, and these vesicles seem to be either fusing with lysosomes to undergo degradation [46] or can fusing with ER-derived pre-peroxisomes to generate the peroxisomes [47]. However, secretion of mitochondrial-derived vesicles into the extracellular space has not previously been described. This is interesting because the current concepts of EV biogenesis do not elucidate any connection with mitochondria.

We detected not only mitochondrial membrane proteins in the EVs, but also many other membrane proteins associated with the plasma membrane and endoplasmic reticulum membrane, that could be another good biomarker candidate. For example, HLA-DR has been used to predict response to anti-PD1/PD-L1 therapy, by determining the presence of HLA-DR in melanoma tissues [48]. In addition, an endoplasmic reticulum membrane protein, Erlin-2, is related to survival of breast cancer by modulating endoplasmic reticulum stress pathways [49]. The diagnostic potential of identifying these proteins in EVs in the circulation would require additional studies.

Multiple critical issues should be considered when interpreting our study. Firstly, it is clear that mitochondrial protein enriched EVs are not released by all cells, as EVs released by MSCs and HEK293 cells have few or no mitochondrial membrane proteins. Therefore, further studies would be required to determine which cells have the capacity to release mitochondrial inner membrane proteins enriched EVs, and the exact mechanisms by which this may happen. Second, we did detect increases in circulating MT-CO2/COX6c in metastatic melanoma patients, and a slight increase in ovarian cancer and breast cancer patients. This specific combination of proteins may, however, not be an ideal biomarker for any malignant disease, as patients with benign ovarian cysts also had increased concentrations of the MT-CO2/COX6c containing EVs in the circulation. However, this study shows that subpopulation of EVs can reflect the presence of disease, and other yet to be described EV subpopulations may have higher sensitivity and specificity to detect disease activity. Third, the sampling methods for blood plasma were the same for disease and healthy controls, and both samples were frozen before further analysis using the sandwich ELISA that we developed internally. Hemolysis could putatively influence the analysis of plasma EVs, but importantly red blood cells do not carry mitochondria [50], and no hemolysis was observed macroscopically. In theory, platelets or white blood cells in the blood sample could release EVs during the sampling, but as the samples were treated the same, and both were frozen prior to analysis, we suggest that the difference between patients and healthy controls is not due to any sampling issue. Fourth, it is challenging to identify appropriate controls for tissue-derived EVs to compare their molecular composition including proteins. Healthy skin or non-malignant naevi tissues from same donor could be appropriate controls from scientific perspective, but it would be very complicated to acquire sufficient amount of tissues. Further, the type of cells and the necrotic/metabolic state of cells are likely to be very different in melanoma vs healthy skin tissue. However, plasma-derived EVs from healthy donors could be used as control EVs, but consist of different components than melanoma tissues, and the EV isolation method would be different. In the current study, we used multiple non-melanoma cell line-derived EVs as comparators rather than controls. It is clear that cell-line derived EVs are isolated with different methodology than tissue-derived EVs, and does not represent tissue microenvironment. To increase the confidence in our findings, we have further compared our tissue proteomics data with published EV proteome data from more than 100 different sources, and using different isolation methods. Lastly, the purity of EVs from human melanoma tissues may not be comparable with cell line-derived EVs, since this is the first demonstration of EV isolation from human tumour tissues where having more dynamic and heterogeneous population of cells.

In summary, we have isolated EVs from melanoma metastatic tissues from patients, and analyzed their surface proteomes, detecting important differences between tissue-derived EVs and classical cell line-derived EVs. Most importantly, mitochondrial membrane proteins were present at high levels in melanoma tissue-derived EVs and represent a subpopulation of EVs. These EVs can be detected in increased concentrations in the plasma of melanoma patients, but also in patients with ovarian- or breast cancer. In addition, we found that certain subpopulations of EVs harbour both active mitochondrial proteins and classical EV markers, suggesting that the classical EV production pathways and the mitochondrial EV pathways may be interacting, which is a new paradigm in EV biology.
