# Supplementary material for: Mitochondrial protein enriched extracellular vesicles discovered in human melanoma tissues can be detected in patient plasma
Source: J Extracell Vesicles. 2019 Aug 27;8(1):1635420. doi: 10.1080/20013078.2019.1635420 (PMC6719261; doi:10.1080/20013078.2019.1635420)
Supplement: Supplemental Material [file ZJEV_A_1635420_SM0358.zip › ZJEV A 1635420_Supplementary/Supplemental Figures-JEV_2nd Revision.docx]

**
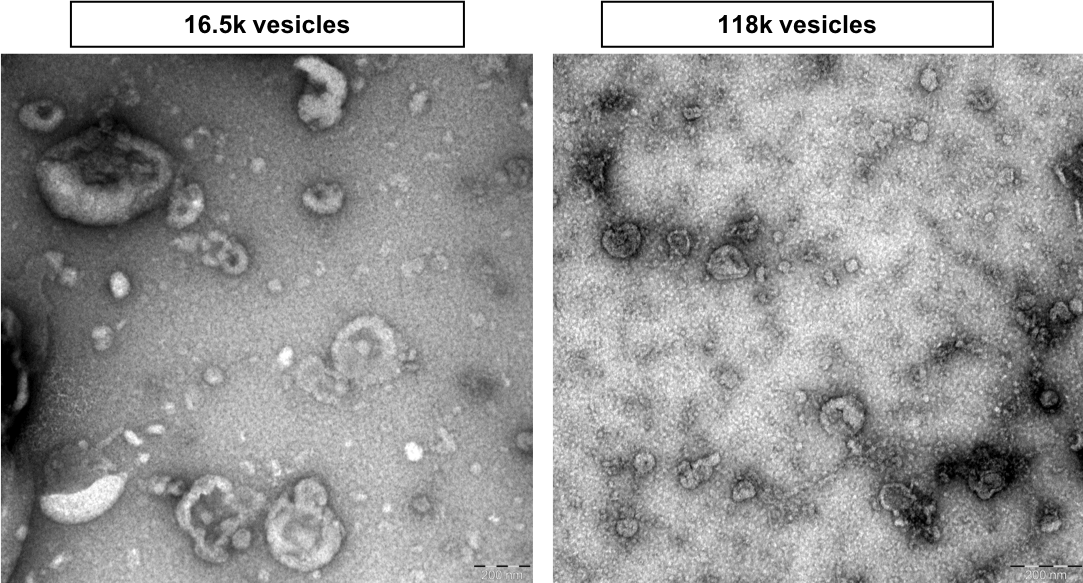
**

**Figure S1. Electron microscopic images of lager and smaller vesicles from melanoma metastases.**

**
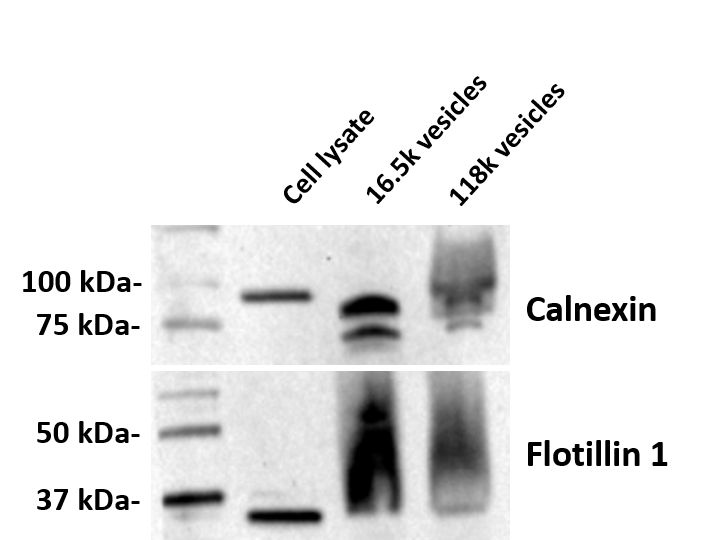
**

**Figure S2. Western Blot of lager and smaller vesicles from melanoma metastases with Calnexin and Flotillin-1.**

**
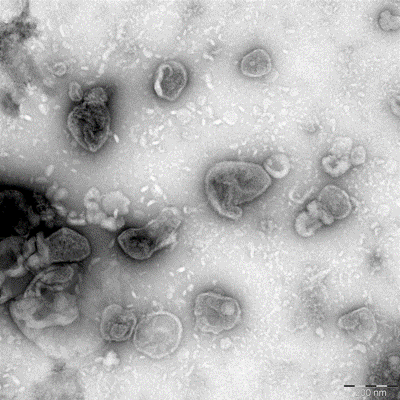
**

**Figure S3. Electron microscopic images of EVs from melanoma metastases after iodixanol density gradient.**

**
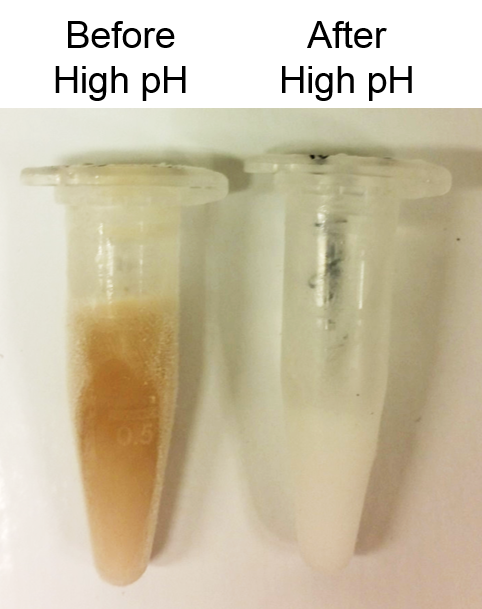
**

**Figure S4. Image of EVs before and after high pH treatment.** Brown color of melanin on EVs were clearly disappeared after high pH treatment.


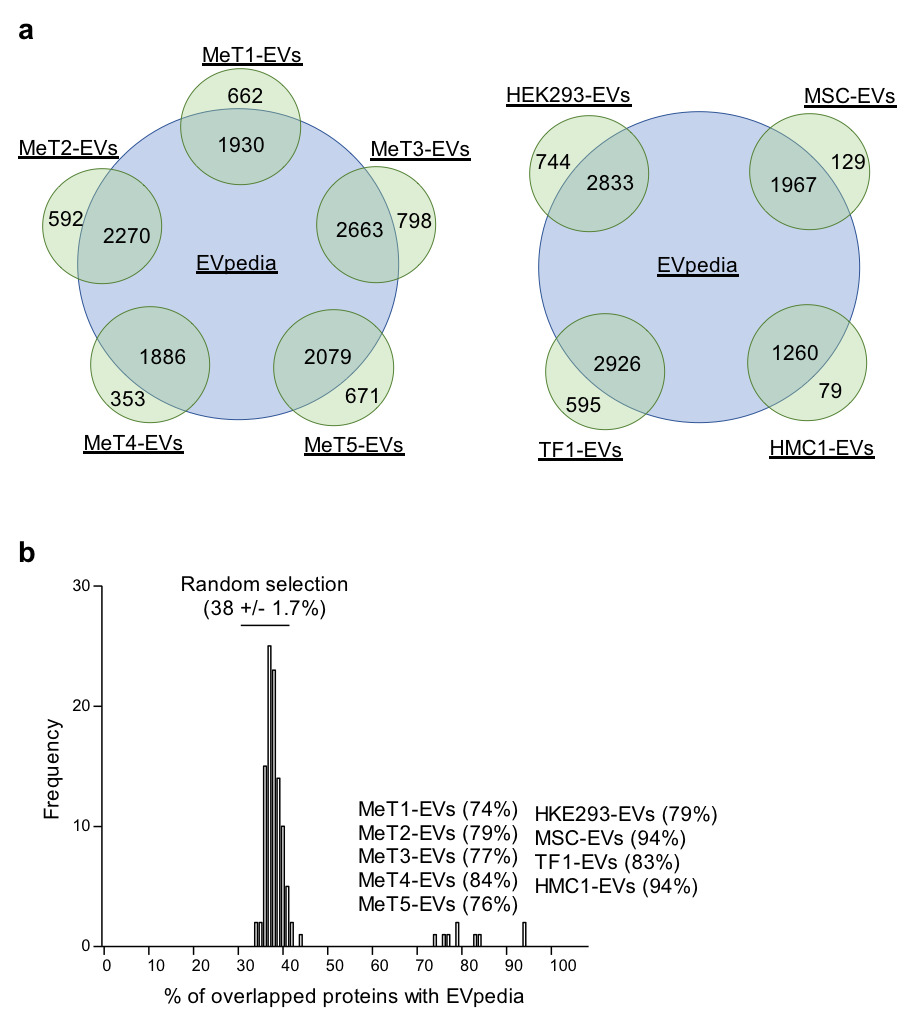


**Figure S5. Comparison with an EV database.** (**a**) Identified EV proteins were well overlapped with the EVpedia. (**b**) The overlapping percentage with EVpedia was higher in EVs compared to randomly selected proteins.


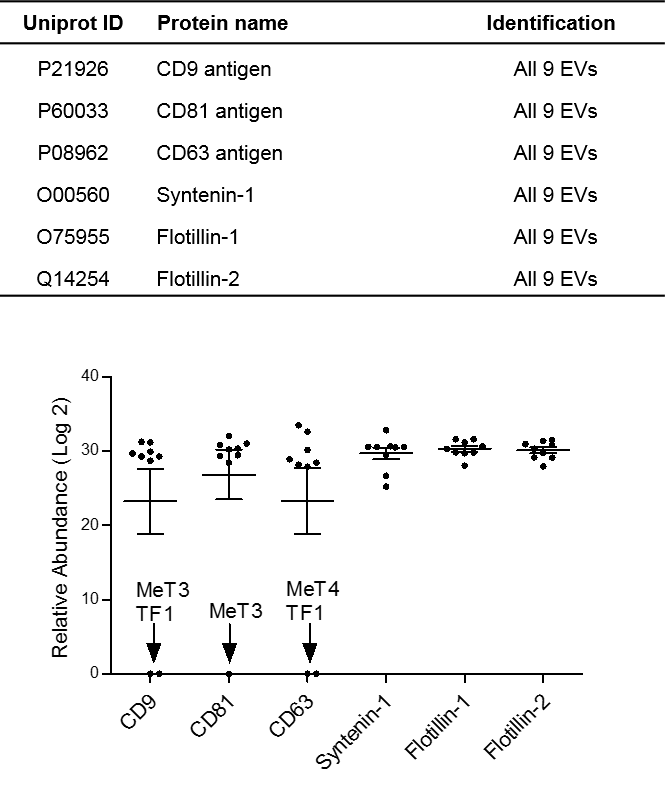


**Figure S6. EV marker identification.** Classical EV membrane marker proteins were identified in all five EV samples with similar abundance.


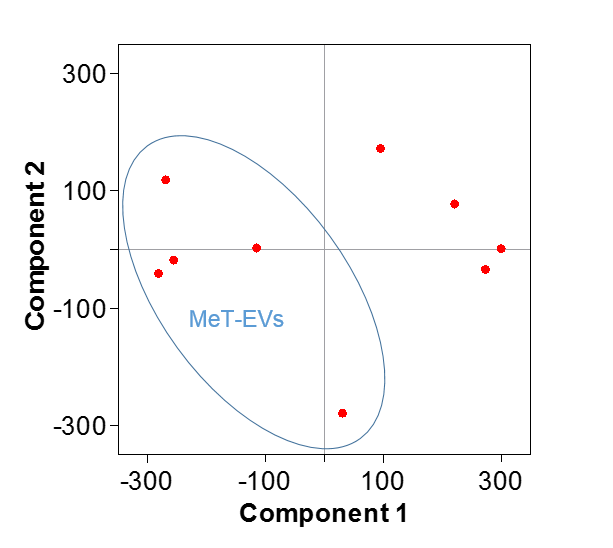


**Figure S7. Principal component analysis of 9 EV proteome.**


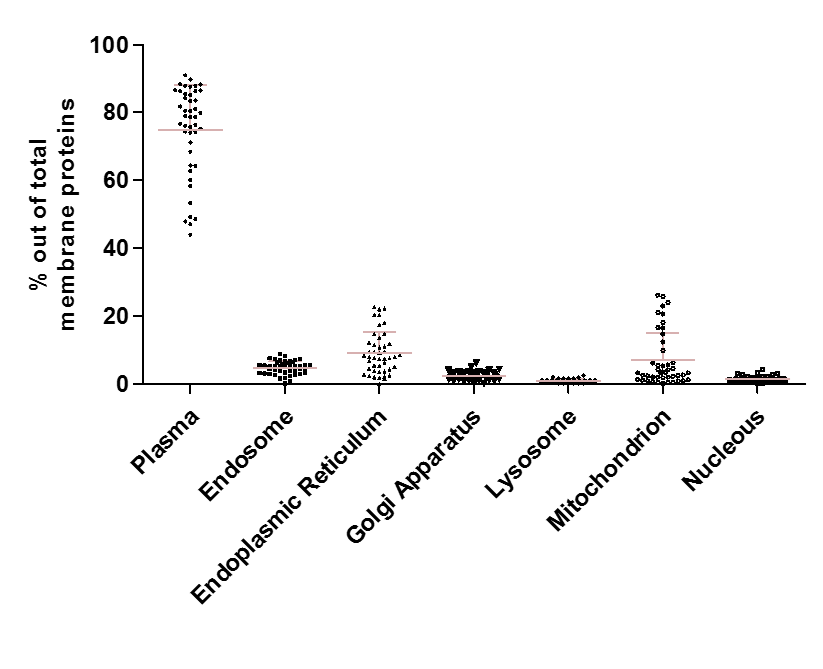


**Figure S8**. **Proportion of organelle membrane proteins.** The percentages of each origin of membrane proteins were calculated from 43 datasets in EVpedia. Localization was obtained from Uniprot and primary localization was used for analysis.


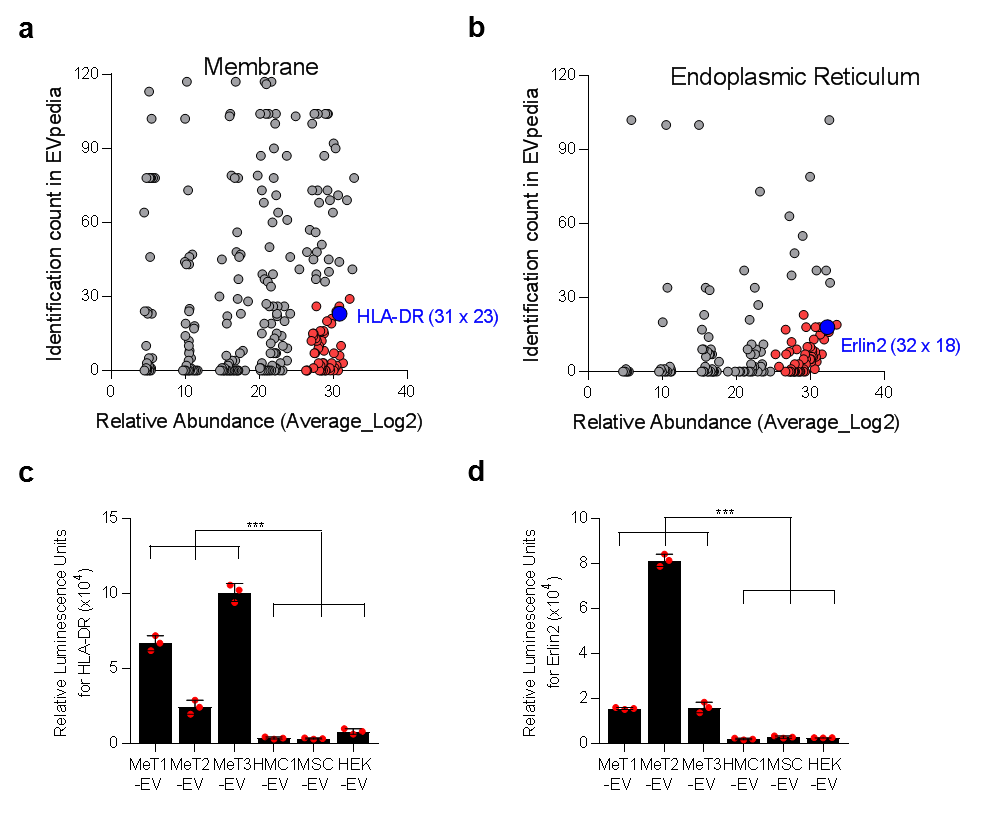


**Figure S9. Validation of proteins by ELISA**. Plasma membrane proteins (**a**) and endoplasmic reticulum membrane proteins (**b**) were plotted with their relative abundance from a mass spectrometry analysis and their identification count from the EVpedia database. Blue color are the final candidates for validation. IC; identification count. HLA-DR (**c**) and Erlin2 (**d**) were experimentally validated with direct ELISA. Data are presented as the mean ± SD. ****p* < 0.001.


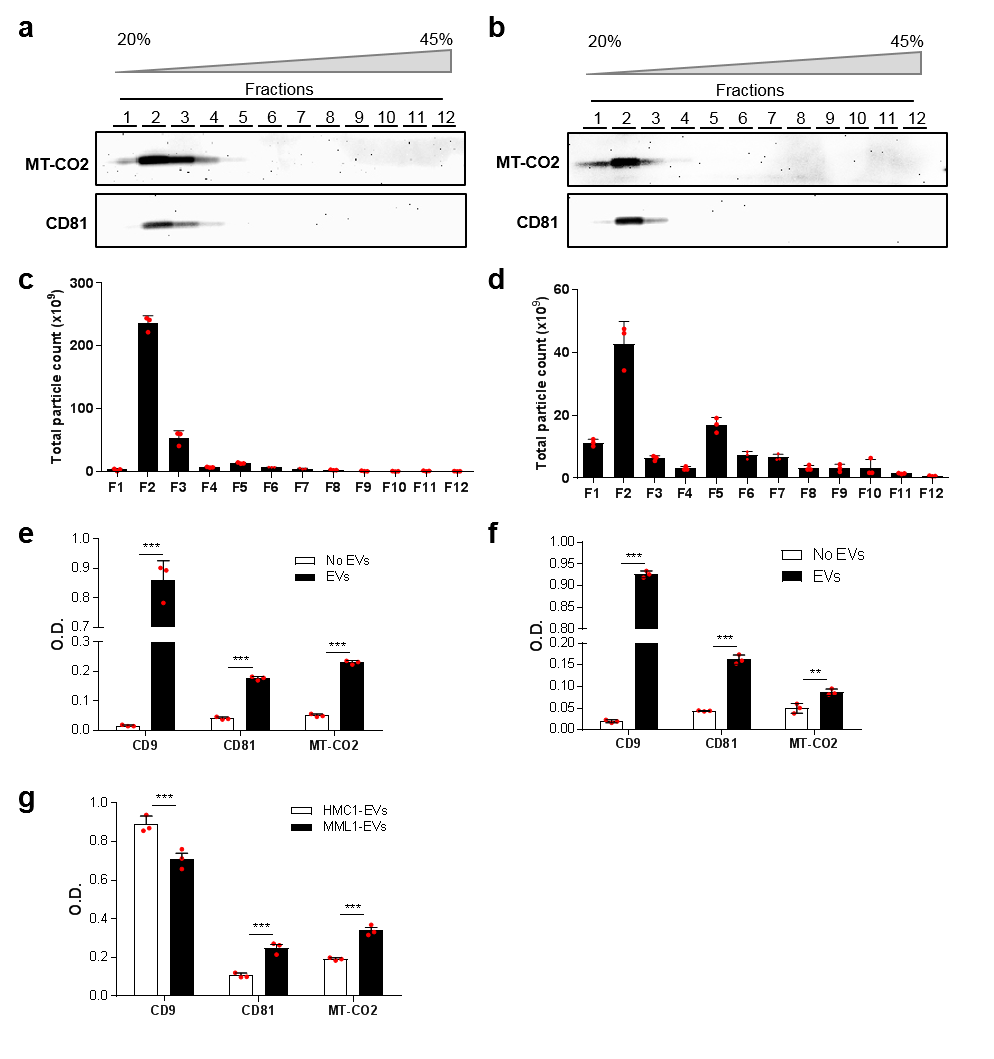


**Figure S10. Mitochondrial MT-CO2 protein on the surface of EVs.** EVs derived from MML1 (**a**) and HMC1 (**b**) were subjected to OptiPrep density gradient purification. MT-CO2 and CD81 expression in each fraction were visualized by Western blot. Particle numbers for each fraction from MML1-EVs (**c**) and HMC1-EVs (**d**) were determined by nanoparticle tracking analysis. Surface expression level of CD9, CD81, and MT-CO2 on MML1-EVs (**e**) and HMC1-EVs (**f**) was measured by direct ELISA. (**g**) The surface expression level was compared between MML1-EVs and HMC1-EVs. Data are presented as the mean ± SD. ***p* < 0.01, ****p* < 0.001.


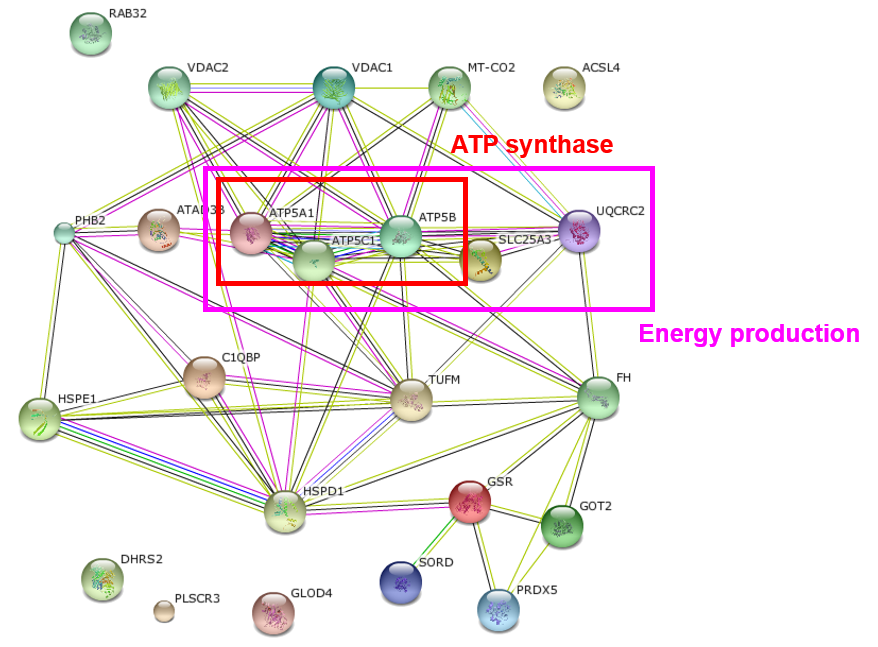


**Figure S11. Protein-protein interaction network of mitochondrial proteins on MTCO2-EVs.** Identified mitochondrial proteins, including ATP synthase subunits and energy production machinery, interacted with each other.

**
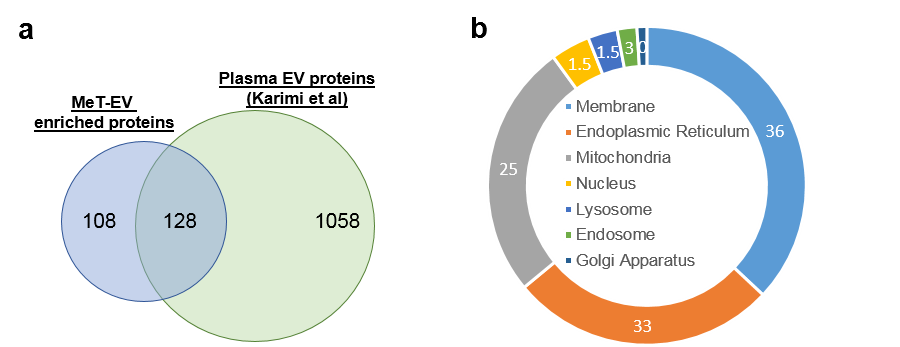
**

**Figure S12. Comparison of melanoma specific proteins with plasma EV proteome.** (a) Venn diagram comparing MeT-EV enriched proteins and plasma EV proteins from Karimi et al. (b) Percentage of subcellular localization of 128 overlapped proteins.


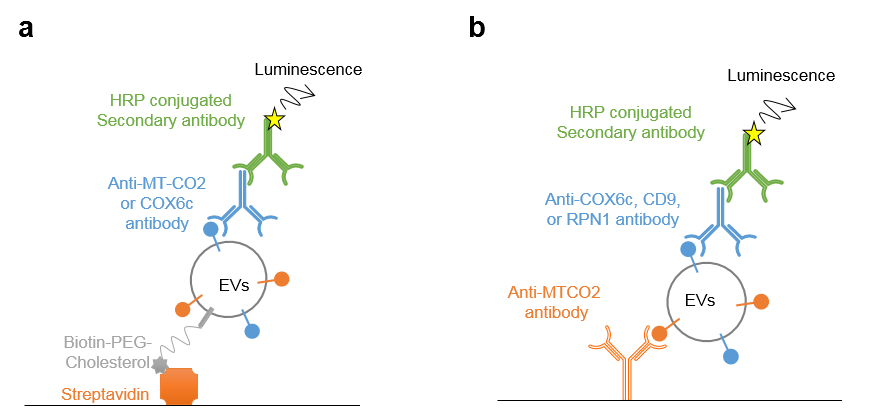


**Figure S13. Schematic illustration of the sandwich ELISA.**  (a) Biotin-PEG-Cholesterol was used to capture EVs, and MT-CO2 or COX6c antibodies were used for detection. (b) MT-CO2 antibody was coated on the plate to capture EVs, and COX6c, CD9, or RPN1 antibodies were used for detection.


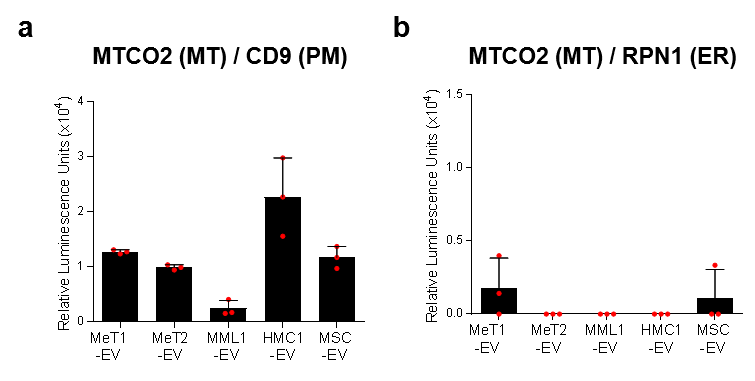


**Figure S14. Sandwich ELISA.** The sandwich ELISA system, MT-CO2/CD9 (a) or MT-CO2/RPN1 (b) combination was validated with melanoma tissue-derived EVs and non-melanoma-derived EVs. Data are presented as the mean ± SD.

**
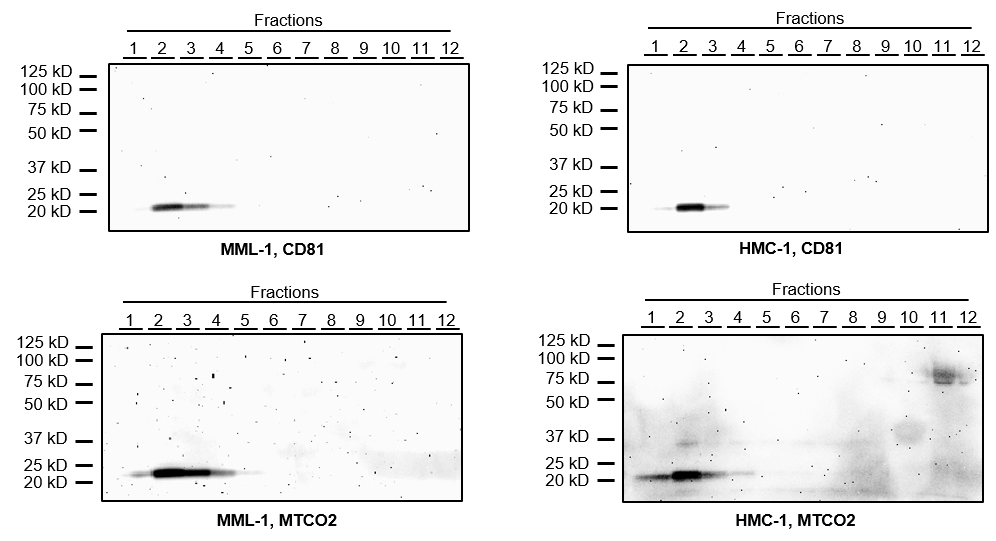
**

**Figure S15.** Full gel image of Western blot analysis.
